# Multiparametric Magnetic Resonance Imaging, Autoimmune Hepatitis, and Prediction of Disease Activity

**DOI:** 10.1002/hep4.1687

**Published:** 2021-02-23

**Authors:** Katherine Arndtz, Elizabeth Shumbayawonda, James Hodson, Peter J. Eddowes, Andrea Dennis, Helena Thomaides‐Brears, Sofia Mouchti, Matt D. Kelly, Rajarshi Banerjee, Stefan Neubauer, Gideon M. Hirschfield

**Affiliations:** ^1^ Centre for Liver and Gastrointestinal Research National Institute for Health Research (NIHR) Birmingham Liver Biomedical Research Centre Birmingham United Kingdom; ^2^ University Hospitals Birmingham National Health Service (NHS) Foundation Trust Birmingham United Kingdom; ^3^ Perspectum Ltd Oxford United Kingdom; ^4^ NIHR Nottingham Biomedical Research Centre Nottingham University Hospitals NHS Trust and University of Nottingham Nottingham United Kingdom; ^5^ Toronto Centre for Liver Disease University Health Network Toronto ON Canada

## Abstract

Noninvasive monitoring of disease activity in autoimmune hepatitis (AIH) has potential advantages for patients for whom liver biopsy is invasive and with risk. We sought to understand the association of multiparametric magnetic resonance imaging (mpMRI) with clinical course of patients with AIH. We prospectively recruited 62 patients (median age, 55 years; 82% women) with clinically confirmed AIH. At recruitment, patients underwent mpMRI with Liver*MultiScan* alongside clinical investigations, which were repeated after 12‐18 months. Associations between iron‐corrected T1 (cT1) and other markers of disease were investigated at baseline and at follow‐up. Discriminative performance of cT1, liver stiffness, and enhanced liver fibrosis (ELF) to identify those who failed to maintain remission over follow‐up was investigated using the areas under the receiver operating characteristic curves (AUCs). Baseline cT1 correlated with alanine aminotransferase (Spearman’s correlation coefficient [*r*_S_] = 0.28, *P* = 0.028), aspartate aminotransferase (*r*_S_ = 0.26, *P* = 0.038), international normalized ratio (*r*_S_ = 0.35 *P* = 0.005), Model for End‐Stage Liver Disease (*r*_S_ = 0.32, *P* = 0.020), ELF (*r*_S_ = 0.29, *P* = 0.022), and liver stiffness *r*_S_ = 0.51, *P* < 0.001). After excluding those not in remission at baseline (n = 12), 32% of the remainder failed to maintain remission during follow‐up. Failure to maintain remission was associated with significant increases in cT1 over follow‐up (AUC, 0.71; 95% confidence interval [CI], 0.52‐0.90; *P* = 0.035) but not with changes in liver stiffness (AUC, 0.68; 95% CI, 0.49‐0.87; *P* = 0.067) or ELF (AUC, 0.57; 95% CI, 0.37‐0.78; *P* = 0.502). cT1 measured at baseline was a significant predictor of future loss of biochemical remission (AUC, 0.68; 95% CI, 0.53‐0.83; *P* = 0.042); neither liver stiffness (AUC, 0.53; 95% CI, 0.34‐0.71; *P* = 0.749) nor ELF (AUC, 0.52; 95% CI, 0.33‐0.70; *P* = 0.843) were significant predictors of loss of biochemical remission. *Conclusion:* Noninvasive mpMRI has potential to contribute to risk stratification in patients with AIH.

## Graphical abstract

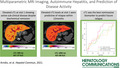

**Abbreviations**
AASLD: American Association for the Study of Liver Diseases
AIH: autoimmune hepatitis
ALT: alanine aminotransferase
APRI: serum aspartate aminotransferase/platelet ratio index
AST: aspartate aminotransferase
AUC: area under the receiver operating characteristic curve
CI: confidence interval
cT1: iron‐corrected T1
ELF: enhanced liver fibrosis
IgG: immunoglobulin G
INR: international normalized ratio
IQR: interquartile range
LS: liver stiffness
MELD: Model of End‐Stage Liver Disease
mpMRI: multiparametric MRI
MRE: magnetic resonance elastography
MRI: magnetic resonance imaging
NHS: National Health Service
NIHR: National Institute for Health Research
PDFF: proton density fat fraction
ULN: upper limit of normal
VCTE: vibration‐controlled transient elastography

Autoimmune hepatitis (AIH) is an orphan chronic liver disease characterized by parenchymal inflammation, the presence of serum autoantibodies, and response to immunosuppression. Liver biopsy is used to support initial diagnosis and exclude alternative/comorbid etiologies. Successful resolution of hepatic inflammation leads to improved clinical outcomes; however, normalization of serum liver enzymes, such as alanine aminotransferase (ALT), does not always exclude underlying residual hepatic inflammation.^(^ ^1^ ^)^ Treatment goals in AIH focus on complete biochemical response (normalization of ALT and immunoglobulin G [IgG]) as well as prevention of disease relapses.^(^ ^2^, ^3^ ^)^

While not necessarily universally practiced because of patient and clinician reluctance,^(^ ^4^ ^)^ clinical guidelines historically recommended repeat on‐treatment histologic assessment by percutaneous liver biopsy to validate the complete resolution of histologic inflammation and to aid in long‐term therapeutic management considerations.^(^ ^2^, ^3^, ^5^ ^)^ However, liver biopsy is associated with significant potential for sampling error, risk of complications from the procedure itself, and significant interobserver variability^(^ ^6^, ^7^, ^8^ ^)^ and is therefore unpopular with patients and some clinicians.^(^ ^4^ ^)^ Thus, recent American Association for the Study of Liver Diseases (AASLD) clinical guidelines recommend biopsy only at diagnosis and treatment stop.^(^ ^2^ ^)^ This reduction in histologic monitoring (using biopsy) has resulted in a need for an alternative, objective, noninvasive means of assessing disease progression.

In keeping with the need to resolve hepatic inflammation and maintain long‐term remission is the clinical imperative to offer tailored therapeutic intervention with the lowest immunosuppression burden. In addition to significant variation in clinical management of AIH across individual clinicians,^(^ ^9^ ^)^ 38%‐93% of patients achieve complete biochemical remission,^(^ ^10^ ^)^ up to 80% of patients experience a disease flare,^(^ ^11^ ^)^ and up to 50% of patients develop cirrhosis.^(^ ^12^ ^)^ A United Kingdom autoimmune hepatitis audit suggested that targeted therapeutic normalization of serum ALT at specific time points may not be an accurate longer term outcome marker and confirmed the clinical and diagnostic need for better noninvasive surrogate markers.^(^ ^13^ ^)^

AASLD guidelines^(^ ^2^ ^)^ include guidance concerning the use of noninvasive techniques, such as serum‐based biomarker panels, vibration‐controlled transient elastography (VCTE) with FibroScan (Echosens, Paris, France), magnetic resonance elastography (MRE), and acoustic radiation force impulse imaging (ARFI) for the assessment of disease progression/regression in AIH. VCTE has been recommended for use in AIH after at least 6 months of treatment^(^ ^2^, ^14^ ^)^ due to the confounding influence of hepatic inflammation on the metric.^(^ ^14^ ^)^ Furthermore, despite showing utility as potential markers of disease, current guidelines do not recommended the use of those fibrosis‐based serum‐based biomarker panels (such as FibroTest,^(^ ^15^ ^)^ serum aspartate aminotransferase [AST]/platelet ratio index [APRI],^(^ ^16^ ^)^ fibrosis‐4 index [FIB‐4],^(^ ^17^ ^)^ and the enhanced liver fibrosis [ELF] test^(^ ^18^ ^)^) because their true utility in AIH is currently unknown.

Multiparametric magnetic resonance imaging (mpMRI) can generate quantitative biomarkers that have demonstrated clinical utility in the assessment of liver disease. One such example is Liver*MultiScan*, a noninvasive noncontrast technology that uses postprocessing of MRI images to combine the assessment of liver fat (using proton density fat fraction [PDFF]),^(^ ^19^ ^)^ iron (using T2*),^(^ ^20^, ^21^ ^)^ and fibroinflammatory disease using iron‐corrected T1 (cT1) relaxation maps. cT1 is an MRI metric that has been shown to correlate with composites of fibrosis and inflammation,^(^ ^22^, ^23^, ^24^ ^)^ be predictive of clinical outcomes,^(^ ^25^, ^26^ ^)^ and to have low interobserver variability and high repeatability over time and across scanners.^(^ ^27^, ^28^ ^)^ cT1 has also been shown to change rapidly with treatment response (early signal of efficacy) and so has utility in both the initial assessment of liver disease as well as in monitoring treatment and improvement, even in the first weeks of treatment in nonalcoholic steatohepatitis.^(^ ^29^ ^)^ However, this technology has not been comprehensively investigated in AIH as a predictor of disease progression and failure to maintain remission.

The aim of this study was to assess the utility of cT1 in a real‐world cohort of patients with AIH followed over time. Our objective was to identify whether we could use this novel imaging technology in patients with resolved biochemistry to predict those patients who would fail to maintain remission. This has the potential to inform a preemptive change in treatment to reduce the likelihood of disease progression. The performance of this technology was also compared to existing noninvasive markers of liver disease.

## Patients and Methods

This study was funded by a National Institute for Health Research (NIHR) grant as an academic collaboration between the University of Birmingham, University Hospitals Birmingham National Health Service (NHS) Trust, and Perspectum Ltd. Local ethical approval was gained through the National Research Ethics Service, West Midlands (Black Country, reference WM/14/0010) along with appropriate data sharing, confidentiality, and collaboration agreements. The study was registered with the International Standard Randomized Controlled Trial Number Registry (ISRCTN39463479) and was NIHR project number 15912. The principles identified in the 1975 Declaration of Helsinki and in Good Clinical Practice were observed throughout the study. All patient identifiable information was kept securely and encrypted within the servers at the study site.

Patients were recruited from a dedicated secondary/tertiary autoimmune liver disease clinic. Patients were being treated for AIH; additional prescreening for inclusion included review of a patient’s working clinical diagnosis and confirmation by the lead investigator (K.A.) that patients met minimum International Autoimmune Hepatitis (IAIH) criteria for the diagnosis of AIH.^(^ ^30^ ^)^ These patients were established on therapy for at least 12 months with no clinical plan at the start of evaluation to alter therapeutic management during the observation period. The standard treatment approach for these patients per our unit practice is initial treatment with prednisone 20‐30 mg per day with tapering starting after 4‐8 weeks and introduction of azathioprine at 1‐2 mg/kg starting at 4 weeks.^(^ ^31^ ^)^ Corticosteroids are generally used for at least 12‐18 months, and azathioprine (or equivalent) most frequently long term. Variations to this approach are highly individualized.

All patients were able to give informed consent to participate in the study. Patients were excluded if they were unable or unwilling to give consent, if they had any contraindications to the study procedures (such as pregnancy or non‐MRI‐compatible implants), if there was any clinical doubt as to the underlying etiology of their liver disease, or if there was evidence of current overt hepatic decompensation (such as encephalopathy or gross ascites).

Patients were assessed on two visits, which were 12‐18 months apart. On each visit, patients underwent noninvasive assessment (including clinical details, medication history, and clinical events), blood analysis (including full blood count, clotting, inflammatory markers, renal function, and liver tests), ELF testing (Siemens Healthineers, Germany), liver stiffness (LS) assessment (FibroScan; Echosens, Paris, France), and mpMRI (Liver*MultiScan*). LS assessment was performed by trained certified operators and deemed valid if 10 valid readings were obtained with an interquartile range (IQR) <30%. Probe choice was based on the FibroScan machine automatic probe selection tool. Where possible, all procedures were completed on the same day after a 4‐hour fast. A 21‐day window was allowed for completion of all procedures.

In this study, loss of remission was defined as an increase in ALT level above the upper limit of normal (ULN) (>41 IU/L).^(^ ^3^ ^)^ Portal hypertension was defined by the presence of at least one of the following: varices, ascites, splenomegaly, and/or low platelet count. Cirrhosis was defined as an irregular liver edge on ultrasound and/or the presence of portal hypertension; elastography readings were not included in this definition as these may have reflected underlying inflammation rather than cirrhosis.

**FIG. 1:**
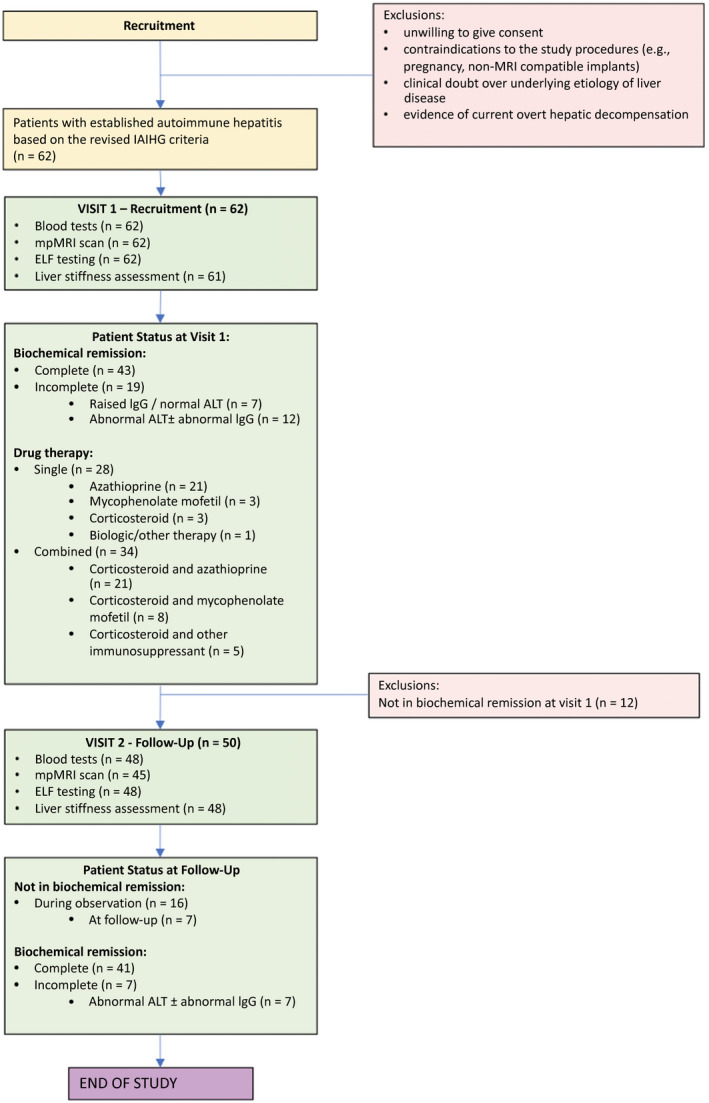
The identification, baseline, and active study procedure followed in this project.

AASLD guidelines define the goal of treatment in AIH to be complete biochemical remission along with normal tissue examination.^(^ ^5^ ^)^ As this study represented a real‐world clinical cohort, protocol histologic assessment was not an approved component within the study; biochemical resolution was used as a surrogate marker for response to treatment. A summary of the procedure followed in this study is show in Fig. 1.

**FIG. 2:**
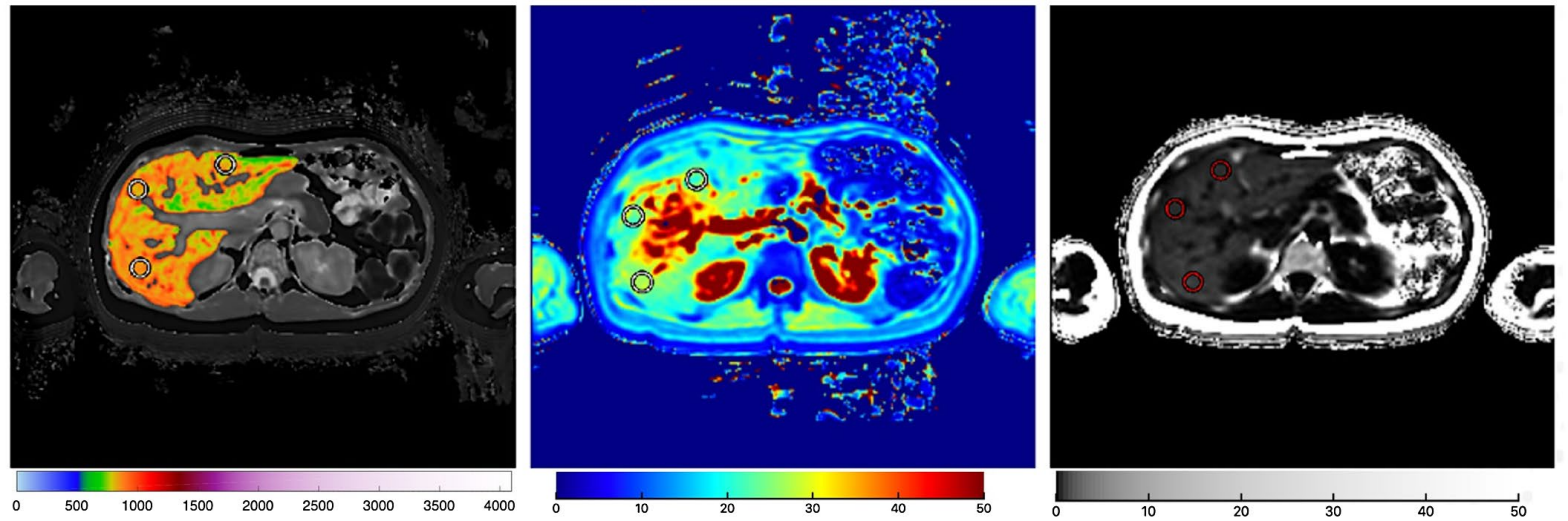
Example images of liver cT1 (left), T2* (middle), PDFF (right) maps in a patient with AIH. Manually placed regions of interest are shown; for cT1, a semi‐automatic liver segmentation map is shown.

The mpMRI scanning protocol was installed, calibrated, and phantom tested on one 3 Tesla Siemens Verio MRI scanner (Siemens Healthcare GMBH, Erlangen, Germany) at University Hospitals Birmingham, and all scans for the study were conducted using the same scanner. Four single‐transverse slices were captured through the liver centered on the porta hepatis. Anonymized MR data were analyzed off‐site using Liver*MultiScan* software (Perspectum Ltd., United Kingdom) by image analysts trained in abdominal anatomy and artefact detection. For T2* and PDFF maps, three 15‐mm diameter circular regions of interest were selected on the transverse maps to cover a representative sample of the liver parenchyma. cT1 maps of the liver were delineated into whole‐liver segmentation maps using a semiautomatic method. cT1 IQR, a measure of the spread of cT1 values across the liver that gives information on disease heterogeneity, was also extracted from the whole‐liver segmentation maps. The mpMRI analysis was completed by investigators blinded to the clinical data and risk grouping. The mpMRI metrics as reported for each patient scan are illustrated in Fig. 2.

### Statistical Analyses

Continuous variables were reported as median and range, categorical variables were reported as frequency and percentage, and confidence intervals (CIs) were reported at the 95% level. Comparisons between patients with and without baseline biochemical remission were performed using Mann‐Whitney U tests for continuous variables and Fisher’s exact tests for nominal variables.

Correlations between all surrogate markers (LS, APRI, ELF, Model for End‐Stage Liver Disease [MELD] score, and international normalized ratio [INR]) with median cT1 and cT1 IQR measured at baseline and follow‐up were then assessed using Spearman’s correlation coefficient (*r*_S_). Given the significant time interval with intervening therapeutic treatment, the amount of historic histologic fibrosis was unlikely to reflect the current clinical situation and thus was not included in the statistical analysis for this study.

Analyses to predict failure to maintain remission occurring during the follow‐up period were then performed after excluding those patients not in biochemical remission at baseline. Overall predictive accuracy of the individual surrogate biomarkers collected at baseline (cT1, cT1 IQR, LS, and ELF) to discriminate future loss of biochemical remission was estimated using the area under the receiver operating characteristic curves (AUCs). Markers found to be significantly predictive were then further assessed using univariable binary logistic regression models to quantify the relationships with failure to maintain remission.

AUCs were also used to investigate the ability of the change over time in the biomarkers to discriminate between patients that did and did not fail to maintain remission during follow‐up. Statistical analyses were performed using R version 3.5.3,^(^ ^32^ ^)^ with *P* < 0.05 deemed to be indicative of statistical significance throughout.

## Results

### Demographics

A total of 62 patients were consecutively consented and recruited to the study. The median age at recruitment was 55 years (range, 22‐80 years), and the majority of patients were women (82%) and of Caucasian ethnicity (89%; Table 1). The median time from liver biopsy to visit 1 was 4.7 years (range, 1.0‐16.6 years), and while all 62 patients with AIH had undergone previous liver biopsy, only 30 full histology reports were available to view. Of these, two (7%) showed no fibrosis, 12 (40%) mild fibrosis, seven (23%) moderate, two (7%) severe, and one (3%) confirmed established cirrhosis.

**Table 1:** Patient demographics at baseline (visit 1) showing patient characteristics, drug therapy, blood panel, and noninvasive liver assessment results as well as the significant difference between those in biochemical remission versus those who are not

Factor	Whole Cohort (n = 62)	Patient Status at Baseline		
		Remission (n = 50)	Not in Remission (n = 12)	*P* Value
Patient characteristics				
Age (years)	55 (22‐80)	55 (22‐80)	46 (27‐73)	0.643
Sex (% female)	51 (82%)	40 (80%)	11 (92%)	0.675
Body mass index (kg/m^2^)	28 (18‐41)	28 (18‐39)	28 (22‐41)	0.854
Ethnicity				1.000
Caucasian	55 (89%)	44 (88%)	11 (92%)	
Non‐Caucasian	7 (11%)	6 (12%)	1 (8%)	
Type 1 AIH	61 (98%)	50 (100%)	11 (92%)	0.194
SLA positive (n = 56)	16 (29%)	14 (32%)	2 (17%)	0.475
Drug therapy				
Single‐agent therapy	28 (45%)	24 (48%)	4 (33%)	0.521
Single‐therapy agent (n = 28)				0.038
Azathioprine	21 (75%)	20 (83%)	1 (25%)	
Corticosteroid	3 (11%)	1 (4%)	2 (50%)	
Mycophenolate mofetil	3 (11%)	2 (8%)	1 (25%)	
Other	1 (4%)	1 (4%)	0 (0%)	
Combined‐therapy agent (n = 34)				0.724
Corticosteroid and azathioprine	21 (62%)	17 (65%)	4 (50%)	
Corticosteroid and mycophenolate mofetil	8 (24%)	6 (23%)	2 (25%)	
Corticosteroid and other immunosuppressant	5 (15%)	3 (12%)	2 (25%)	
Blood panel and liver assessment results				
Platelets (×10^9^/L)	212 (40‐352)	221 (40‐352)	159 (45‐324)	0.061
INR	1.0 (0.9‐2.8)	1.0 (0.9‐1.4)	1.2 (1.0‐2.8)	0.012
ALT (IU/mL)	21 (9‐219)	18 (9‐35)	60 (43‐219)	<0.001
AST (IU/L)	24 (12‐193)	22 (12‐38)	61 (39‐193)	<0.001
IgG (g/L)	11.4 (4.1‐27.2)	10.9 (4.1‐27.2)	15.6 (8.6‐26.3)	0.029
Bilirubin (μmol/L)	10 (4‐57)	9 (5‐20)	15 (4‐57)	0.002
MELD	7 (6‐14)	7 (6‐11)	8 (6‐14)	0.021
APRI	0.30 (0.09‐9.97)	0.27 (0.09‐1.80)	0.84 (0.44‐9.97)	<0.001
ELF	9.38 (7.67‐12.67)	9.37 (7.67‐11.62)	10.33 (8.84‐12.67)	0.015
LS (kPa) (n = 61)	6.9 (2.9‐27.7)	6.9 (2.9‐27.7)	7.9 (3.1‐23.4)	0.104
cT1 (milliseconds)	850 (735‐973)	844 (735‐973)	876 (804‐963)	0.155
cT1 IQR (milliseconds)	121 (73‐268)	118 (73‐230)	136 (85‐268)	0.064
Clinical cirrhosis	25 (40%)	19 (38%)	6 (50%)	0.521
Portal hypertension	15 (24%)	9 (18%)	6 (50%)	0.054
Varices/ascites	7 (11%)	5 (10%)	2 (17%)	
Imaging features	8 (13%)	4 (8%)	4 (33%)	

Data are reported as median (range) with *P* values from Mann‐Whitney U tests or as n (%) with *P* values from Fisher’s exact tests. *P* < 0.05 is considered statistically significant.

Abbreviation: SLA, soluble liver antigen.

At baseline, 50 patients (81%; ALT (IU/mL) <41 IU/mL ± IgG <ULN; n = 6 having elevated IgG [range, 17.70‐26.58 g/L]) were classified as being in complete biochemical remission while 12 (19%) had incomplete biochemical response (ongoing abnormal liver tests ± abnormal IgG) (Fig. 1). Of the markers considered at visit 1, there was one missing LS result (technical difficulties). At the baseline visit, 28 (45%) patients were on single‐agent therapy (azathioprine, n = 21; mycophenolate mofetil, n = 3; corticosteroid, n = 3; and biological therapy, n = 1) while 34 (55%) were on combined drug therapy (corticosteroid and azathioprine, n = 21; corticosteroid and mycophenolate mofetil, n = 8; and corticosteroid and other immunosuppressants, n = 5).

Baseline comparisons between those not in biochemical remission (n = 12) and the remainder of the cohort (n = 50) found no significant differences in patient demographics, such as age (*P* = 0.643), sex (*P* = 0.675), or body mass index (*P* = 0.854) (Table 1). The proportions of patients treated with single‐agent therapy were also similar in the two groups (*P* = 0.521). However, within the subgroup of patients on single‐agent therapy, a significant difference in the distribution of drugs used was observed (*P* = 0.038), with patients not in biochemical remission being less likely to be treated with azathioprine but more likely to be receiving corticosteroids or mycophenolate mofetil. Liver assessments, such as INR, ALT, AST, IgG, and MELD, were significantly raised in those not in biochemical remission at baseline, as would be expected. The cohort as a whole had a median cT1 of 850 milliseconds (range, 735‐973 milliseconds), which is above the upper ninety‐fifth percentile value of 763 milliseconds that was observed in a large, healthy, normal population.^(^ ^33^ ^)^ However, neither the cT1 (*P* = 0.155) nor cT1 IQR (*P* = 0.064) was found to be significantly higher in those not in biochemical remission at baseline. LS was also not found to be significantly raised in those not in biochemical remission at baseline (*P* = 0.104), although a significant difference in ELF was detected (*P* = 0.015).

### Correlations of Baseline MRI With Serum Markers of AIH Activity, Disease Severity, and Fibrosis

The correlations of mpMRI markers with commonly used clinical markers of disease severity and fibrosis at baseline can be seen in Table 2. cT1 significantly correlated with markers of disease activity, such as ALT (*r*_S_ = 0.28, *P* = 0.028) and AST (*r*_S_ = 0.26, *P* = 0.038). In addition, cT1 also correlated significantly with surrogate markers of disease severity, such as INR (*r*_S_ = 0.35, *P* = 0.005), MELD (*r*_S_ = 0.32, *P* = 0.020), ELF (*r*_S_ = 0.29, *P* = 0.022), and LS (*r*_S_ = 0.51, *P* < 0.001).

**Table 2:** Correlations (*r*_S_) Between cT1 and cT1 IQR With Blood Test Results and Other Surrogate Markers of Liver Health at Baseline and Follow‐Up

	Baseline (n = 62)		Follow‐Up (n = 45)	
	cT1	cT1 IQR	cT1	cT1 IQR
Correlation with serum liver and liver function tests				
Platelets	−0.09	−0.40	0.17	−0.29
	*P* = 0.499	*P* = 0.001	*P* = 0.281	*P* = 0.054
ALT	0.28	0.18	0.41	0.08
	*P* = 0.028	*P* = 0.171	*P* = 0.005	*P* = 0.594
AST	0.26	0.37	0.45	−0.05
	*P* = 0.038	*P* = 0.003	*P* = 0.002	*P* = 0.736
Bilirubin	0.17	0.49	−0.18	−0.07
	*P* = 0.180	*P* < 0.001	*P* = 0.237	*P* = 0.653
IgG	0.25	0.19	0.22	0.27
	*P* = 0.055	*P* = 0.131	*P* = 0.139	*P* = 0.074
Correlation with surrogate disease severity markers				
LS (n = 61)	0.51	0.52	0.38	0.23
	*P* < 0.001	*P* < 0.001	*P* = 0.010	*P* = 0.127
APRI	0.24	0.46	0.15	0.19
	*P* = 0.058	*P* < 0.001	*P* = 0.327	*P* = 0.207
MELD	0.32	0.23	0.27	−0.12
	*P* = 0.010	*P* = 0.076	*P* = 0.076	*P* = 0.432
ELF	0.29	0.20	0.29	−0.06
	*P* = 0.022	*P* = 0.128	*P* = 0.070	*P* = 0.729
INR	0.35	0.22	0.29	−0.08
	*P* = 0.005	*P* = 0.081	*P* = 0.052	*P* = 0.606

*P* < 0.05 is considered statistically significant.

Similar analyses performed to understand the associations between disease heterogeneity (cT1 IQR) and liver function tests showed significant correlations with platelets (*r*_S_ = −0.40, *P* = 0.001), AST (*r*_S_ = 0.37, *P* = 0.003), and bilirubin (*r*_S_ = 0.49, *P* < 0.001). Moreover, cT1 IQR also correlated significantly with other markers of disease severity, namely LS (*r*_S_ = 0.52, *P* < 0.001 ) and APRI (*r*_S_ = 0.46, *P* < 0.001).

### Disease Monitoring During the Observation Period

Of the 50 patients in biochemical remission, 48 returned for follow‐up (visit 2), of whom 45 had a follow‐up mpMRI (Fig. 1). At follow‐up, cT1 significantly correlated with ALT (*r*_S_ = 0.41, *P* = 0.005), AST (*r*_S_ = 0.45, *P* = 0.002), and LS (*r*_S_ = 0.38, *P* = 0.010) (Table 2).

A total of 16/50 (32%) patients failed to maintain remission during the follow‐up period (interim loss of biochemical remission). Four of the 16 patients had a relapse episode with ALT >3 times ULN (ALT = 302, 206, 448, 340 IU/mL) with an associated rise in IgG, while the remaining 12 patients had milder elevations in ALT and/or IgG. Of the 6 who had elevated IgG at baseline, 4 had an improvement and 2 did not reach complete IgG resolution. No patient developed *de novo* clinical cirrhosis or portal hypertension, and all patients who experienced an ALT rise resulting in loss of biochemical remission during the study follow‐up period subsequently received changes to their medication regime, with 1 patient starting *de novo* corticosteroid therapy and the remaining having their dosage increased. Investigations were performed to determine the sensitivity of biomarker to interim loss of biochemical remission. As such, the changes in the levels of biomarkers between the two patient visits were calculated, and the discriminative ability of these with respect to interim loss of biochemical remission was quantified. This found patients with an interim loss of biochemical remission to have a significantly greater increase in cT1 over the follow‐up period, with an AUC of 0.71 (95% CI, 0.52‐0.90; *P* = 0.035). Changes in LS (AUC, 0.68; 95% CI, 0.49‐0.87; *P* = 0.067), cT1 IQR (AUC, 0.57; 95% CI, 0.36‐0.78; *P* = 0.457), or ELF (AUC, 0.57; 95% CI, 0.37‐0.78; *P* = 0.502) were not found to be significantly discriminative with respect to interim loss of biochemical remission.

### Follow‐Up and Prediction of Future Failure to Maintain Remission

**FIG. 3:**
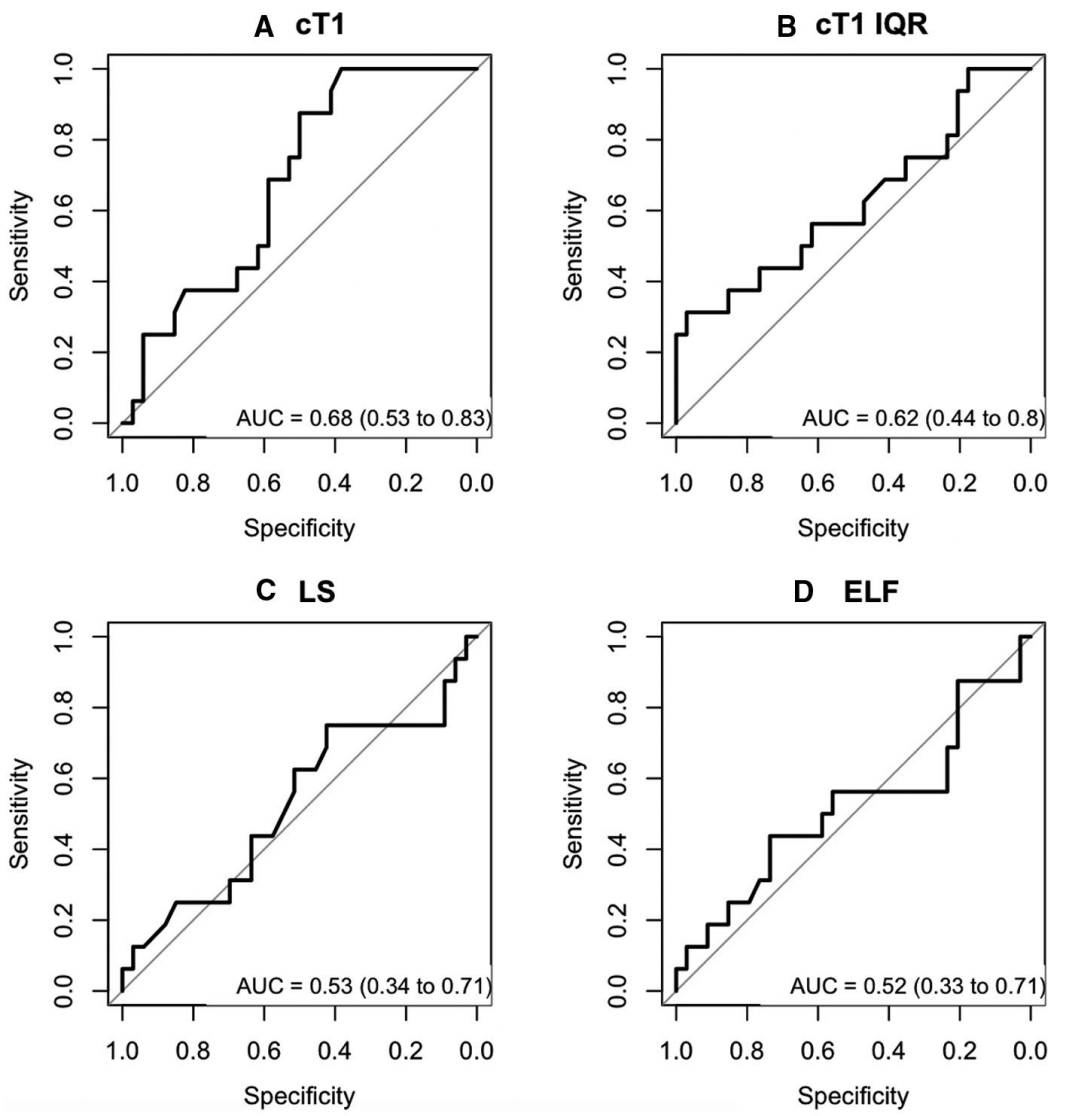
AUCs showing the predictive capability of surrogate biomarkers. AUCs are shown for (A) cT1, (B) cT1 IQR, (C) LS, and (D) ELF to identify those who were in biochemical remission at baseline but will experience disease progression and failure to maintain remission at follow‐up.

The predictive accuracy of baseline measures with respect to future failure to maintain biochemical remission (i.e., interim loss of biochemical remission) was then assessed (Fig. 3). Baseline cT1 was found to be a significant predictor of future loss of biochemical remission, with an AUC of 0.68 (95% CI, 0.53‐0.83; *P* = 0.042) and sensitivity 1.0, specificity 0.38, positive predictive value 0.43, and negative predictive value 1.0 for a cT1 Youden cutoff of 814 milliseconds. However, ELF (AUC, 0.52; 95% CI, 0.33‐0.71; *P* = 0.843), LS (AUC, 0.53; 95% CI, 0.34‐0.71; *P* = 0.749), and cT1 IQR (AUC, 0.62; 95% CI, 0.44‐0.80; *P* = 0.170) were not found to be significant predictors of future failure to maintain remission.

**FIG. 4:**
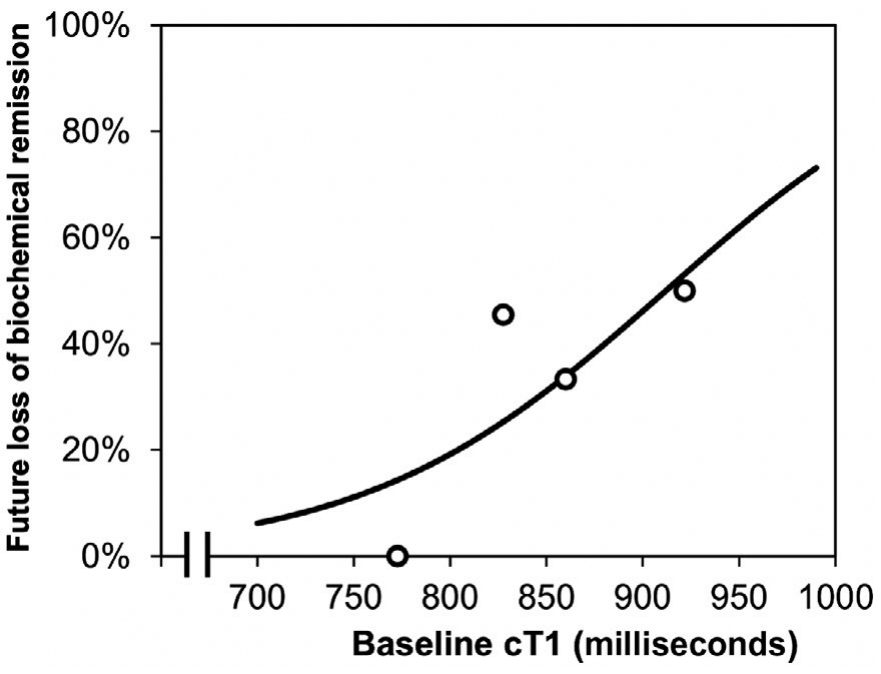
Associations between baseline cT1 and rate of remission loss in patients with complete biochemical remission at baseline. The trendline is from a univariable binary logistic regression model. Points represent the observed rates of subsequent loss of biochemical remission within quartiles of the distribution and are plotted at the mean of the intervals.

The association between baseline cT1 and interim loss of biochemical remission was then assessed in further detail using a univariable binary logistic regression model. This returned an odds ratio for future failure to maintain remission of 1.14 per 10‐millisecond increase in baseline cT1 (95% CI, 1.01‐1.28; *P* = 0.036), which is visualized in Fig. 4. None of the 12 patients with cT1 <800 milliseconds at baseline had a subsequent loss of biochemical remission. The model estimated that a cT1 of 800 milliseconds at baseline was associated with a 19% risk of failure to maintain remission, which increased to 76% for a cT1 of 1,000 milliseconds.

## Discussion

Although relatively rare, AIH is nevertheless associated with ongoing morbidity and mortality. Clinical practice and treatment guidelines frequently diverge as a reflection of disease heterogeneity, lack of registered treatments, challenges in agreeing standards of care, a reluctance on the part of clinicians and patients to use liver biopsy, and an increasing recognition of treatment burden for patients. We sought to explore the utility of mpMRI imaging in a real‐world cohort of patients with AIH followed over 1 year.

We investigated the correlations between cT1 and other surrogate markers of disease used clinically (INR, ALT, AST, IgG, MELD, ELF, LS, and APRI score). Results showed baseline cT1 to be significantly correlated with ALT and AST as well as with other surrogate markers (LS, MELD, ELF, and INR). As these markers are used to infer liver status, these associations reflect the ability of cT1 to characterize liver tissue within a real‐world cohort, consistent with AIH standard of care.

LS (measured by VCTE) has been shown to correlate with biochemical remission and regression of fibrosis; however, VCTE is affected by hepatic inflammation.^(^ ^14^ ^)^ The utility of this technique to inform patient management is therefore limited to use after at least 6 months of treatment.^(^ ^2^, ^14^ ^)^ In terms of fibrosis staging and diagnosis of cirrhosis, MRE has been shown to outperform VCTE, conventional MRI, and fibrosis scoring systems (FIB‐4, APRI).^(^ ^34^, ^35^ ^)^ Nevertheless, MRE can be influenced by therapy, liver inflammation, and hepatic fibrosis. Moreover, MRE is not able to differentiate between untreated and treated patients with AIH,^(^ ^2^ ^)^ which may be important because interface and lobular hepatitis cause hepatocyte apoptosis and fibrogenesis in untreated patients. ARFI has shown comparable utility with VCTE in predicting both fibrosis and cirrhosis^(^ ^36^ ^)^ and has shown potential to assess manifestations of portal hypertension^(^ ^37^ ^)^; however, it has been shown to overestimate hepatic fibrosis.^(^ ^38^ ^)^ mpMRI has the potential to provide noninvasive, objective, and accurate metrics for whole‐liver tissue characterization assessment, yielding clinically meaningful information.^(^ ^39^ ^)^ Disease heterogeneity across the liver is a known characteristic of AIH,^(^ ^2^ ^)^ and noninvasive characterization of this is potentially important as it cannot be evaluated by existing tests used clinically in AIH.^(^ ^40^ ^)^ Therefore, results from this study showing the utility of using cT1 IQR to quantify the heterogeneity of fibroinflammation disease within the liver volume provide added useful information not available today.

In terms of monitoring the impact of interim disease flaring on liver state, results from this study showed that cT1 was a marginally better marker of change in disease severity (AUC, 0.71) when compared to LS (AUC, 0.68) and ELF (AUC, 0.57). Of particular importance to clinical management was the superior performance of cT1 in discriminating those patients who experienced disease progression and failed to retain biochemical remission during the follow‐up period. Subsequent exploratory analyses using univariate logistic regression models showed the prognostic ability of cT1 to predict future disease progression and loss of biochemical remission in patients who start with biochemical remission; patients with cT1 of 800 milliseconds at baseline had a 19% risk of disease progression leading to loss of biochemical remission; this increased to 76% at 1,000 milliseconds. As LS and ELF did not show similar prognostic capability, these data highlight the potential for cT1 to be used as a risk‐stratification tool informing treatment titration or cessation in patients with complete biochemical response.

With infrequent liver biopsy and given the real‐world practice of our clinic, we did not feel it would be appropriate to include a *de novo* liver biopsy in the study protocol as it deviated from standard of care; however, we acknowledge the limitations this brings to the study. Specifically, the lack of liver histology resulted in the inability to assess the correlations between cT1 and liver histology at both time points in the study. Previous studies have reported strong correlations between MR‐based metrics and histologic measures of fibrosis and inflammation; however, prospective studies pairing mpMRI techniques and biopsy in AIH are justified to enable further understanding of the associations between cT1 and liver inflammation and fibrosis^(^ ^13^ in this population. On correlation analysis, the strengths and significances of associations between cT1/cT1 IQR and other surrogate biomarkers (apart from ALT and AST) were lower when assessed at the follow‐up visit compared to the analysis at baseline. This difference may partly be explained by the reduction in statistical power resulting from the smaller sample size at follow‐up after excluding those patients who were not in biochemical remission at baseline and those that did not undergo mpMRI at follow‐up. The exclusion of those who were not in biochemical remission at baseline may have introduced selection bias to the latter analysis, with the cohort not being representative of that analyzed at baseline. In addition, patients who fail to maintain biochemical remission have been shown to have a higher frequency of cirrhosis and have also shown evidence of portal hypertension. Because 24% had evidence of cirrhosis with portal hypertension, this could have attributed to the frequency of suboptimal response. Therefore, future analyses following the same consistent cohort over time might yield a better understanding of the changes associated with the correlation of these markers over time. Nevertheless, from the results presented above, it is evident that cT1 is capable of characterizing liver fibroinflammatory activity in an orphan disease where there is a growing unmet need due to the decrease in liver biopsy and the variability associated with patient care in clinical practice.

In conclusion, mpMRI demonstrated underlying fibroinflammatory activity as well as the breadth of abnormalities seen within the liver. By demonstrating an ability to identify those who will experience disease progression and loss of biochemical remission, mpMRI has shown promise in the phenotyping and risk stratification of individuals with high‐risk disease who may not be identified using serum biochemistry alone. This proof of concept study identifies mpMRI as a disruptive technology and justifies future prospective clinical trials in this area, potentially in combination with liver biopsy, to fully explore its utility. There is the potential to develop this technology further to aid in clinical decision making, such as to improve identification of patients at risk of flare events and to provide an evidence base for making therapeutic decisions.
